# Positive Response to One-Year Treatment With Burosumab in Pediatric Patients With X-Linked Hypophosphatemia

**DOI:** 10.3389/fped.2020.00048

**Published:** 2020-02-18

**Authors:** Silvia Martín Ramos, Marta Gil-Calvo, Virginia Roldán, Ana Castellano Martínez, Fernando Santos

**Affiliations:** ^1^Hospital Universitario Central de Asturias, Oviedo, Spain; ^2^Hospital Clínico Universitario de Santiago, Santiago de Compostela, Spain; ^3^Hospital Universitario Puerta del Mar, Cádiz, Spain; ^4^Instituto de Investigación Sanitaria del Principado de Asturias, Oviedo, Spain; ^5^Universidad de Oviedo, Oviedo, Spain

**Keywords:** X-linked hypophosphatemia (XLH), rickets, burosumab, FGF23, hypophosphatemia, vitamin D, children

## Abstract

X-linked hypophosphatemia (XLH) causes significant burden in pediatric patients in spite of maintained treatment with phosphate supplements and vitamin D derivatives. Administration of burosumab has shown promising results in clinical trial but studies assessing its effect in the everyday practice are missing. With this aim, we analyzed the response to one-year treatment with burosumab, injected subcutaneously at 0.8 mg/kg every 2 weeks, in five children (three females) aged from 6 to 16 years, with genetically confirmed XLH. Patients were being treated with phosphate and vitamin D analogs until the beginning of burosumab treatment. In all children, burosumab administration led to normalization of serum phosphate in association with marked increase of tubular reabsorption of phosphate and reduction of elevated serum alkaline phosphatase levels. Baseline height of patients, from −3.56 to −0.46 SD, increased in the three prepubertal children (+0.84, +0.89, and +0.16 SD) during burosumab treatment. Growth improvement was associated with reduction in body mass index (−1.75, −1.47, and −0.17 SD, respectively), suggesting a salutary effect of burosumab on physical activity and body composition. Burosumab was well-tolerated, mild local pain at the injection site and transient and mild headache following the initial doses of burosumab being the only reported undesirable side effects. No patient exhibited hyperphosphatemia, progression of nephrocalcinosis, worsening of metabolic control or developed hyperparathyroidism. Mild elevation of serum PTH present at the beginning of treatment in one patient 4 was not modified by burosumab administration. These results indicate that in the clinical setting, beyond the strict conditions and follow-up of clinical trials, burosumab treatment for 1 year exerts positive effects in pediatric patients with XLH without major adverse events.

## Introduction

X-linked hypophosphatemic rickets (XLH) is a monogenic disorder that follows a Mendelian dominant inheritance (OMIM 307800) and it is the most common hereditary form of rickets (1).

XLH is caused by loss of function of the gene *PHEX* (2), a phosphate regulating gene with homologies to endopeptidases, leading to elevated circulating levels of fibroblast growth factor 23 (FGF23), renal wasting of phosphate and defective renal production of 1,25-dihydroxyvitamin D (3).

Although XLH is characterized by a broad phenotypical expression, active lesions of rickets, subsequent bone deformities and disharmonic growth retardation are major manifestations of the disease in the pediatric age. Dental abscesses and mineralization defects in teeth are also common findings (1).

Classical medical treatment of XLH is based on the oral administration of phosphate supplements and 1-hydroxy metabolites of vitamin D, given chronically, at least until the end of body growth (4). This therapy usually heals the active radiological lesions of rickets and improves growth but does not normalize serum phosphate, does not avoid major clinical manifestations of the disease, does not result in the achievement of a normal final adult height and entails the risk of potential undesirable side effects such as nephrocalcinosis or hyperparathyroidism (5, 6).

The development of burosumab, a humanized monoclonal antibody for FGF23 is a promising treatment in patients with XLH (7). Preliminary clinical trials indicate that burosumab improves renal tubular phosphate reabsorption, serum phosphorus levels, linear growth, and physical function and reduces pain and the severity of rickets in children with XLH (8, 9). However, very limited data on pediatric patients with XLH treated with burosumab are available. Thus, to know the efficacy and safety of burosumab treatment in the everyday clinical setting we here present five children with genetically confirmed XLH receiving the drug for more than a year.

## Patients and Methods

Patients younger than 18 years of age with XLH genetically confirmed and on treatment with burosumab for more than a year were identified in three Spanish hospitals. All patients received burosumab subcutaneously at a dose of 0.8 mg/kg every 2 weeks, after an initial dose of 0.4 mg/kg. Phosphate supplements and 1-hydroxy derivatives of vitamin D administration were withdrawn 2 weeks before the beginning of burosumab.

After informed consent, the medical records of these patients were reviewed. The following data were collected from the basal visit, immediately before starting burosumab, and, when available, from each of the outpatient clinic visits during the period of burosumab treatment: height, weight, and clinical manifestations including adverse effects potentially related with the administration of burosumab; fasting serum concentrations of phosphate, alkaline phosphatase, calcium, 1,25-dihydroxyvitamin D and intact parathyroid hormone (PTH); urine calcium/creatinine ratio, tubular phosphate reabsorption (TPR) and tubular maximum reabsorption of phosphate for glomerular filtration rate (TmP/GFR) were calculated. Radiological findings as well as the presence of nephrocalcinosis or dental impairment were also collected.

Somatometric measurements, including height, weight, and body mass index (BMI), were expressed in absolute values and as standard deviation (SD) score of Spanish reference growth charts for age and sex. The profile of the several biochemical variables measured was drawn using absolute values except for alkaline phosphatase whose values were expressed as percentage of the initial one because of the wide dispersion of the results in the different hospitals.

The protocol was approved by *Comité de Ética de la Investigación con Medicamentos del Principado de Asturias (SP no 38/18)*. All subjects gave written informed consent in accordance with the Declaration of Helsinki.

## Results

Table 1 shows baseline characteristics of the patients before starting burosumab treatment. Hypophosphatemia associated to urinary wasting of phosphate, relatively low values of 1,25-dihydroxyvitamin D and elevated alkaline phosphatase levels all support the diagnosis of XLH, confirmed by the mutation found in the *PHEX* gene. Serum concentrations of calcium and 25-hydroxyvitamin D were normal (not shown). Serum intact PTH levels were also normal except in patient 4 who had a mild elevation (upper normal reference value 65 pg/mL).

**Table 1:** Clinical, genetic, and biochemical findings in five children with X-linked hypophosphatemia at the moment of starting treatment with burosumab, after withdrawal of administration of vitamin D derivatives and phosphate supplements at least 2 weeks before.

	**Patient 1**	**Patient 2**	**Patient 3**	**Patient 4**	**Patient 5**
Age (years)	7	6	16	13	13
Sex	Female	Male	Female	Male	Female
*PHEX* gene mutation and zigosity	c.871C>T; p.Arg291Ter Heterozygous	c.670C>T; p.Gln224Ter Hemizygous	c. 1936_?del; p.Asp646_?del Heterozygous	c.1079+1G>A; splicing Hemizygous	c.1735G>A; p.Gly579Arg Heterozygous
Serum phosphate (mg/dL)	2.2	2.6	1.7	2.3	1.7
Serum AP (U/L)	504	425	516	1093	605
Serum 1,25(OH)_2_D (pg/mL)	15.9	36.6	22.0	13.4	23.0
Serum PTH (pg/mL)	34	19	22	83	40
TRP (%)	61	54	32	85	80
TmP/GFR (mg/dL)	0.90	1.40	0.54	1.99	1.36
Height in cm (SD)	119.9 (−0.53)	105.0 (−2.4)	143.5 (−2.89)	129.7 (−3.56)	154.2 (−0.46)
Skeletal findings	Leg bowing Hyperlordosis	Leg bowing	Leg bowing	Leg bowing Scaphocephaly	Leg bowing
Dental abnormalities	No	No	No	Dental abscesses	Dental abscesses

*AP, alkaline phosphatase; PTH, parathyroid hormone; 1,25(OH)_2_D, 1,25-dihydroxyvitamin D; TRP, tubular reabsorption of phosphate; TmP/GFR, tubular maximum reabsorption of phosphate for glomerular filtration rate*.

**Figure 1:**
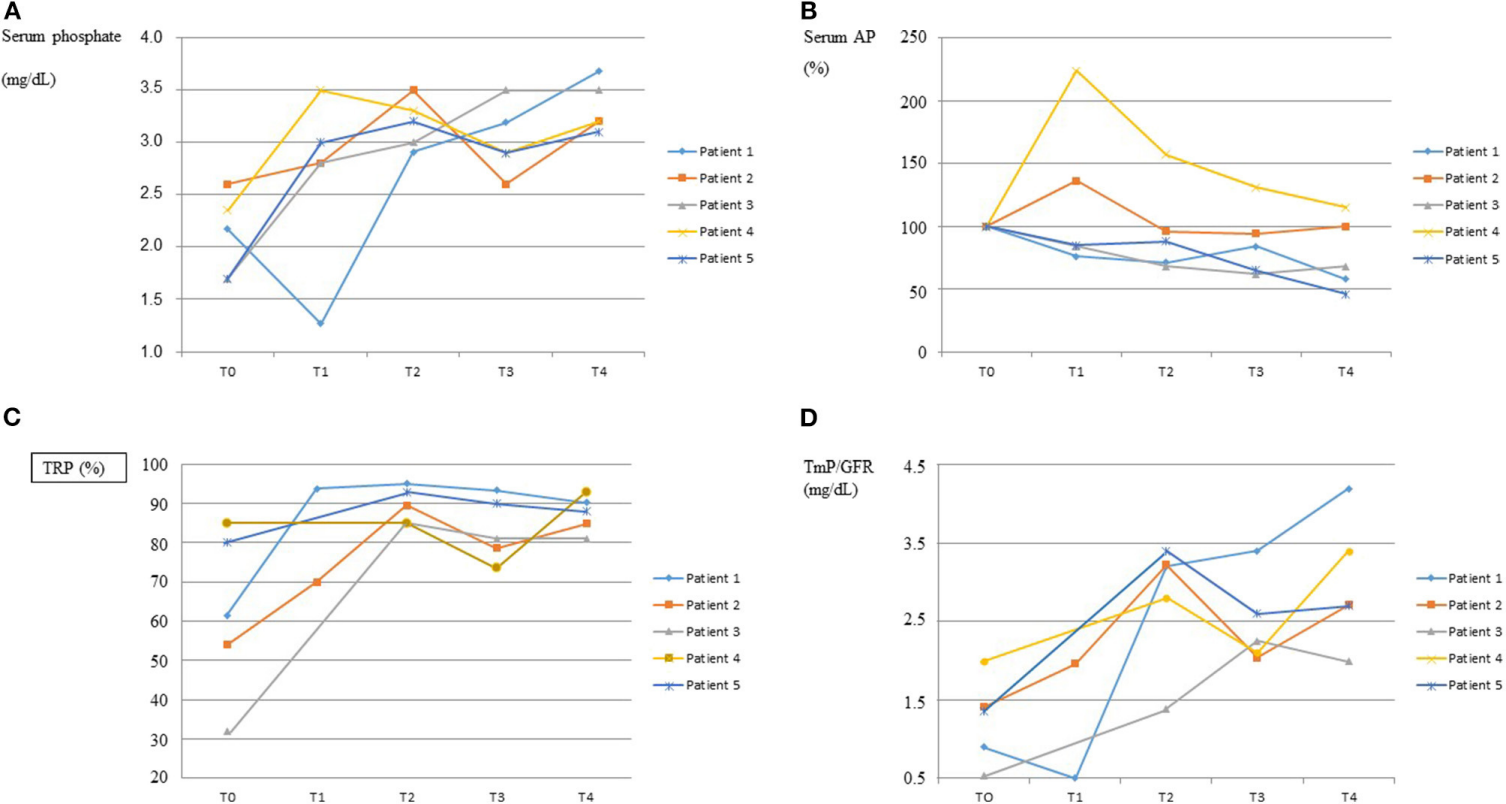
Response to burosumab. The course over time of serum phosphate, serum alkaline phosphatases (AP), tubular reabsorption of phosphate (TRP) and tubular maximum reabsorption of phosphate for glomerular filtration rate (TmP/GFR) are shown in graphs **(A–D)**, respectively, for each patient. On the “x” axis, T0 indicates baseline values, immediately before starting burosumab treatment, and T1–T4 indicate values every 3–4 months on burosumab treatment. X ± SD values for T0 and T4 were as follows. Serum Phosphate (mg/dL): 2.10 ± 0.39 and 3.33 ± 0.24; Serum AP (U/L): 628 ± 267 and 525 ± 419; TRP (%): 62.5 ± 21.3 and 87.4 ± 4.7; TmP/GFR (mg/dL): 1.26 ± 0.53 and 2.99 ± 0.83.

Response to burosumab assessed by the biochemical profiles of serum phosphate, serum alkaline phosphatase and renal reabsorption of phosphate is graphically shown in Figure 1. The dose of burosumab did not need to be modified and was kept at 0.8 mg/kg every 2 weeks as originally planned. Serum phosphate normalized in all patients, in patient 1 after a transient worsening of hypophosphatemia immediately following the first burosumab dose. The normalization of serum phosphate was associated to greater renal phosphate reabsorption as shown by increases in TRP and TmP/GFR.

Growth of patients is shown in Table 2. Height improved in three out of the five children and remained unchanged in two who were pubertal females of 13 and 16 years of age. Weight and BMI decreased, particularly in those children who exhibited greater increase in height SDS, or remained unchanged.

**Table 2:** Effect of 1 year burosumab treatment on growth in five children with X-linked hypophosphatemia.

	**Patient 1**	**Patient 2**	**Patient 3**	**Patient 4**	**Patient 5**
Height in cm (SD)	130.0 (0.31)	115.4 (−1.51)	144.5 (−2.92)	136.0 (−3.4)	155.6 (−0.76)
Height Δ (SD)	+0.84	+0.89	−0.03	+0.16	−0.30
Weight in kg (SD)	33 (0.79)	27.2 (0.37)	46 (−1.22)	43.7 (−1.14)	53.5 (−0.02)
Weight Δ (SD)	−0.97	−0.39	−0.11	−0.07	−0.18
BMI in kg/m^2^ (SD)	19.53 (0.84)	20.42 (1.66)	22.03 (0.14)	23.63 (0.64)	22.27 (0.37)
BMI Δ (SD)	−1.75	−1.47	−0.08	−0.17	−0.02

*BMI, body mass index; Δ, SD change during burosumab treatment*.

As for the unsuitable effects of burosumab, no patient developed new radiological lesions, nephrocalcinosis or dental problems during the period of burosumab treatment. The patients had been receiving conventional treatment of XLH and did not have active radiological lesions of rickets. As for nephrocalcinosis, it was already present in one patient before starting treatment with burosumab and the kidney ultrasounds did not worsen during burosumab treatment. No patient developed new findings of nephrocalcinosis after 1 year of burosumab treatment. Likewise, no adverse effects potentially related with burosumab were reported except for mild local pain at the injection site (1 patient) and transient and mild headache following the initial doses of burosumab (1 patient). Patient 4 had elevation of PTH at the beginning of treatment. None of the four remaining patients developed hyperparathyroidism, serum PTH concentrations increasing in all of them but within the normal range.

## Discussion

This manuscript supports the efficacy and safety of burosumab administered to pediatric patients with XLH in the everyday clinical setting, beyond the strict monitoring conditions of clinical trials (8, 9). This is important because, as stated in a recently published evidence-based guideline for the diagnosis and management of XLH (10), recommendations on the use of burosumab cannot be conclusive because the available information is based on the results of trials testing the drug in children with severe XLH (10). We here report that treatment with burosumab for 1 year at a subcutaneous dose of 0.8 mg/kg/14 days results in normalization of serum phosphate, decrease of serum alkaline phosphatase, elevation of serum 1,25-dihydroxyvitamin D, and marked improvement of tubular reabsorption of phosphate in five pediatric patients with genetically confirmed XLH after interruption of classical treatment with phosphate supplements and vitamin D derivatives. It is of note that TRP (%) of patients 4 and 5 were within the expected range for a child with normal serum phosphate concentrations but TRP should be much higher (above 90–95%) in the presence of marked hypophosphatemia such as that found in these two patients, below 2.5 mg/dl in both.

Burosumab induced a positive effect on growth in three out of the five children, as demonstrated by a marked increase of height SDS at the end of the 1-year treatment period. In these patients the amelioration of height was noticeably higher than that found by Carpenter et al. (8) in their trial after 64 weeks of treatment. XLH exerts a marked adverse effect on growth (11) and final adult height of pediatric patients with XLH is usually low, even if they have received conventional treatment since diagnosis (12) or growth hormone (13) at infancy or early childhood. Our findings indicate that the growth promoting action of burosumab does not occur in growth retarded pubertal XLH patients, regardless its positive effects on phosphate metabolism. The two patients (patient 1 and 2) with the greater height SD increase during burosumab treatment were those of younger age (7 and 6 years), suggesting the convenience of early administration of burosumab, before puberty. It also supports the assumption that the impairment of growth in XLH is not entirely dependent on hypophosphatemia and rachitic bone lesions (11, 14) so that growth retardation may persist in spite of improvement in mineral metabolism. The characterization of growth response to burosumab needs to be investigated in further studies involving greater number of patients and longer follow-ups.

It is interesting to note that BMI markedly decreased in patients 1 and 2. These patients underwent the greatest improvement in height and, although, unfortunately, our study does not provide quantitatively measured objective data on musculoskeletal function (15), this positive effect on weight and body composition likely resulted from the higher physical activity of children, a fact disclosed by the patients' parents in the clinical interviews. Likewise, families reported the interruption of frequent oral doses of phosphate as an appreciable benefit linked to the new treatment. Burosumab also exerted a beneficial effect on radiological osseous abnormalities although no specific score to quantify bone deformity and/or legs' bowing was used.

Burosumab was well-tolerated and no serious undesirable effects were detected. Headache and local injection-site reactions reported by one of our patients have been notified in the literature in approximately 60% of children treated with burosumab (16). No patient exhibited hyperphosphatemia, progression of nephrocalcinosis, worsening of metabolic control or developed hyperparathyroidism. Mild elevation of serum PTH present at the beginning of treatment in patient 4 was not modified by burosumab administration.

In summary, this study shows the efficacy and safety of burosumab administered for 1 year in the clinical management and follow-up of pediatric patients with XLH. Additional studies are necessary to assess the influence of burosumab on the long-term outcome of these patients and to define the criteria for its use.

## Data Availability Statement

All datasets generated for this study are included in the article/supplementary material.

## Ethics Statement

The studies involving human participants were reviewed and approved by Comité de Ética de la Investigación con Medicamentos del Principado de Asturias (SP n° 38/18). Written informed consent to participate in this study was provided by the participants' legal guardian/next of kin. Written informed consent was obtained from the minor(s)' legal guardian/next of kin for the publication of any potentially identifiable images or data included in this article.

## Author Contributions

SM designed the data chart, obtained patient's information from the medical records, analyzed the data, drew the figures, and contributed to writing the manuscript. MG-C, VR, and AC obtained patients' information from the medical records, revised and corrected the data presentation, and the manuscript. FS designed the study, revised and corrected the data presentation and contributed to writing the different versions of the manuscript.

### Conflict of Interest

The authors declare that this study received funding from Kyowa Kirin. The funder had the following involvement with the study: financial support for FS teaching and research activities.
